# Treatment of Olive Mill Wastewater through Integrated Pressure-Driven Membrane Processes

**DOI:** 10.3390/membranes10110334

**Published:** 2020-11-11

**Authors:** Aldo Bottino, Gustavo Capannelli, Antonio Comite, Camilla Costa, Raffaella Firpo, Anna Jezowska, Marcello Pagliero

**Affiliations:** Department of Chemistry and Industrial Chemistry, University of Genoa, via Dodecaneso 31, 16141 Genova, Italy; bottino@chimica.unige.it (A.B.); gustavo.capannelli@gmail.com (G.C.); camilla.costa@unige.it (C.C.); firpolella00@gmail.com (R.F.); aniajez@yahoo.com (A.J.); marcello.pagliero@unige.it (M.P.)

**Keywords:** olive mill wastewater, membrane separation process, microfiltration, reverse osmosis, water recovery

## Abstract

The disposal of wastewater resulting from olive oil production (olive mill wastewater, OMW) is a major issue for olive oil producers. This wastewater is among the most polluting due to the very high concentration of organic substances and the presence of hardly degradable phenolic compounds. The systems proposed for OMW treatment are essentially based either on conventional chemical-physical, biological and thermal processes, or on membrane processes. With respect to conventional methods, membrane processes allow to separate different species without the use of chemicals or heat. This work deals with the use of the integrated pressure-driven membrane processes for the treatment of OMW. They consist of a first stage (microfiltration, MF) in which a porous multichannel ceramic membrane retains suspended materials and produces a clarified permeate for a second stage (reverse osmosis, RO), in order to separate (and concentrate) dissolved substances from water. Laboratory scale experiments with different small flat sheet RO membranes were first carried out in order to select the most appropriate one for the successive bench scale tests with a spiral wound module having a large membrane surface. The aim of this test was to concentrate the dissolved substances and to produce water with low salinity, chemical oxygen demand (COD), and reduced phytotoxicity due to a low content of phenolic compounds. The trend of the permeate flux and membrane retention as a function of the volume concentration ratio was investigated. The influence of OMW origin and its aging on the membrane performance was also studied.

## 1. Introduction

Olive oil mill wastewater (OMW) is a by-product of the olive oil extraction process produced seasonally in a large quantity. Niaounakis and Halvadakis in their book [1] estimated a generation of OMW in the range of 10–30 million m^3^/year in 2006 and we should expect that since then, its quantity has increased in accordance with the increase in world olive oil consumption, which from 2006 to 2019 has grown from about 2.6 to 2.97 million tons [2,3].

### 1.1. OMW Composition

The OMW consists mainly of olive fruit vegetation water (more than 50% of the fruit) and water added during the extraction process. The composition of OMW is affected by the variety and ripeness of the olives and by the system used for their processing (pressure or centrifugation mills). For example, the centrifugation step in three-phase olive mill processing, the most common olive oil extraction system, generates an amount of OMW more than two times higher than that of olive oil produced. An average OMW composition can be given as 83.2% of water, 1.8% of inorganic salts and 15% of organic constituents, among which 7.5% of sugars [4]. OMW is characterized by a low pH, a high electrical conductivity and a chemical oxygen demand (COD), which can be as high as 200 g/L. The three-phase process (3P) generates the greatest amount of OMW, about 1–1.2 m^3^/tons of olives, while the two-phase process generates the least amount, about 0.085–0.1 m^3^/tons of olives. The batch-pressing process produces about 0.4–0.6 m^3^/tons of olives of OMW [5]. Nevertheless, all the three types of OMW are highly pollutant. Due to the presence of several organic compounds, among which there is a phenolic fraction, untreated OMW has broad-spectrum toxicity against bacteria, plants and animals [6], which implies treatment and environmental problems. However, phenols presence in OMW makes this problematic by-product (wastewater) a potential source for recovery of precious antioxidants. For the abovementioned reasons, OMW treatment systems are not only supposed to be flexible and efficient in reducing COD and salinity, they also should be a viable alternative for recovery of high added value phenolic compounds. 

### 1.2. OMW Membrane-Based Treatment Processes

The systems proposed for OMW treatment are essentially based either on conventional biological, chemical, physicochemical and thermal processes [7,8,9] or advanced membrane processes [10,11,12,13,14,15,16]. The latter, especially pressure-driven processes (microfiltration (MF), ultrafiltration (UF), nanofiltration (NF) and reverse osmosis (RO)), offer several advantages over traditional technologies, mainly in terms of low energy consumption, no additive requirements and no phase change, and thus, the possibility to preserve the original characteristics of treated effluents. 

Gebreyohannes et al. [17] in 2016 reviewed both the literature and patents about the application of integrated membrane technologies for OMW treatment and they highlighted the polarization and fouling problems occurring in the pressure-driven membrane processes, which are mainly related to the particular composition of the OMW (e.g., solids, pectins, etc.). Again in 2016, Pulido [18] reviewed in detail the open literature on the application of membrane technologies in OMW treatment as well as on the main obstacles for their cost-effective utilization, namely the related fouling problems. He highlighted the need for a pretreatment before the integrated membrane process to limit the fouling phenomena and to achieve more stable operating permeate fluxes.

Typically, the proposed integrated pressure-driven membrane processes are based on the combination of steps for the removal of suspended solids (e.g., microfiltration or ultrafiltration) and of a second step aimed at the pollutant concentration and clean water recovery (e.g., nanofiltration and/or reverse osmosis). A fractionation of the pollutants contained in the OMW is technically feasible [19,20] but the application of such a process scheme composed by several steps of MF and UF with different molecular weight cut-off (MWCO), NF and RO is very expensive and often quite sophisticated for its implementation into small and medium olive mills. 

Membrane processes have been applied to all the three types of OMWs (Table 1). The content of total suspended solids (TSS) and others minor components such as fats and pectins makes imperative a feed pretreatment before the NF or RO processes. Considering that the pH for the types of OMWs is similar, the electrical conductivity (EC) reflects the concentration of organic electrolytes and salts. The two-phase process shows the lowest electrical conductivity or solid residue. On the other hand, the two-phase process generates a solid, known as alperujo, which is a very pollutant waste to handle since it contains most of the organic compounds that in the three-phase process are released in the wastewater [5]. 

Cassano et al. [9] studied the application of UF polymeric membranes (MWCO between 4 and 10 kDa) and they observed a flux decrement up to 50% over 300 min operating time. The best performing membrane was made of regenerated cellulose. The flux recovery after cleaning with an alkaline detergent at 40 °C was claimed enough to recover the initial water flux. In any case, the raw OMW was subjected to preventive microfiltration step at 0.2 micron. Garcia Castello et al. [11] studied the combination of MF, NF followed by an osmotic distillation. In the MF step a 0.2 µm membrane was used and a strong flux decrease was observed without any tendency of stabilization. The cleaning procedure was carried out by using a concentrated alkaline solution of 20 g/L NaOH at 40 °C for 30 min followed by tap water rinsing. An irreversible fouling was observed with a loss of flux of about 50%. The flux reduction in the NF membrane (Nadir N30F spiral-wound membrane module) was about 35% after about 1 h operation at a volume reduction factor of about 3. The NF membrane after cleaning with 1g/L of NaOH as done for MF completely recovered its initial water flux. From the cited investigations it seems that although UF underwent severe fouling the initial flux could be recovered in most of the cases by a chemical alkaline cleaning procedure. 

Bazzarelli et al. [28] proposed an integrated membrane process based on a MF/NF and osmotic distillation and membrane emulsification. For the MF step, a 0.14 μm ceramic membrane was used and the good results in the MF flux stability were ascribed to an acidification step at pH 1.8 and a subsequent filtration on a stainless steel filter [29]. The chemical cleaning protocol was still based on the use of an alkaline detergent at 40 °C for 30 min. Chemical physical pretreatments before the integrated pressure-driven membrane processes were studied in order to improve the performance of the integrated membrane process. Pulido et al. [30] applied a pretreatment based on a Fenton process, then directly followed by NF. Nevertheless, the direct application of tighter membrane processes (NF or RO) after a physical chemical secondary treatment can lead to cake formation on the membrane surface as reported for RO membranes [31].

Recently, the possibility of using a water-ethanol mixture for the extraction of polyphenols and their purification by integrated membrane processes was explored [32]. Although it opens up an interesting perspective, additional investigations should be carried out in order to define the quality of the reverse osmosis permeate and its ethanol content. With the aim of recovering valuable polyphenols, most of the studies investigated the integration of ultrafiltration (UF) and (NF). De Almeida et al. [22] showed that despite the combination of UF and NF, the COD and total phenols removal can be 83.3 and 93.1%, respectively. Despite the interesting results, the quality of the permeate water is still far from being disposed in the sewage under the parameters imposed by the legislation. Therefore, to meet the current disposal regulations a further treatment or filtration step of the NF permeate is clearly necessary. 

Another integration scheme relied on the direct use of RO instead or in addition to the NF. Tundis et al. [33] showed the recovery and classification of polyphenols by using a MF step on a 0.1 µm TiO_2_ membrane followed by a NF step and a RO step based on a membrane typically applied to brackish water. Although a flux decay was observed for all the membrane filtrations, as the aim of the work was about the characterization of the polyphenols in the concentrate, the quality of the final NF and RO permeates was not assessed by the authors. Zagklis et al. [34] in a recent paper mentioned the design of a full system based on UF/NF/RO integrated with adsorption steps and solid-liquid extraction with the aim of recovering the polyphenolic fractions from both OMW and other types of phenolic containing wastewater (e.g., grape marc and olive leaves). Coskun et al. [35] in their lab scale study proposed centrifugation as a primary step followed by UF and finally by RO. Their study was exclusively focused on the rejection performance of the different membranes. Petrotos et al. [36] studied some relevant operational parameters on a pilot scale, a process integrating MF followed by a NF (or open RO) and then by RO using tubular membranes. 

The problem of OMW is clearly urgent from the point view of its environmental impact and the technological solution that requires it to be simple, cost-effective and reliable, especially in countries where the size of working olive mills is still small. Integrated pressure-driven processes, which include RO as a final step, should enable the production of a permeate water of sufficient quality not only for its safe discharge into sewage, but also for any kind of reuse into a farm or olive oil production process. 

Membrane processes were shown to be very effective in the treatment of numerous industrial effluents and wastewaters. However, their successful application depends on the proper choice of process configuration and process conditions, and these are the focus of the experimental study presented here. In this work, two consecutive pressure-driven membrane processes, namely microfiltration and reverse osmosis, are proposed for OMW treatment in order to obtain a RO permeate composed of water with a low salinity, COD, and reduced phytotoxicity due to very low content of phenolic compounds, which are retained and concentrated by the RO membrane. To this aim, laboratory scale tests were first carried out with small flat sheet RO membrane samples in order to select the most suitable membrane for the successive pilot scale investigation with a spiral wound element with a large membrane surface area. The high concentrations of suspended materials in OMW imposed the use of microfiltration as a pretreatment system for the RO, in order to avoid plugging of the feed spacer of the spiral wound elements. Ceramic multichannel elements with excellent thermal stability and chemical resistance to withstand severe cleaning cycles were used for microfiltration in order to easily remove particulates that can foul the membrane or plug the channels. This work deals with practical aspects and problems connected to the concentration of large volumes of OMW with MF/RO pilot plants and to OMW storage that were not investigated enough in the literature. 

## 2. Materials and Methods

Since characteristics of OMW may differ significantly from mill to mill, OMWs from three different olive mills, two located in Liguria and one in Tuscany, were employed. The names of these mills cannot be revealed for confidentiality reasons and a generic code composed of letters and numbers will be used to identify the three types of OMWs. OMWs were first stored in reservoir tanks to allow sedimentation of a large part of suspended materials and separation of a supernatant fluid, which was filtered through a filter bag with an opening of 200 µm prior to microfiltration. 

**Figure 1:**
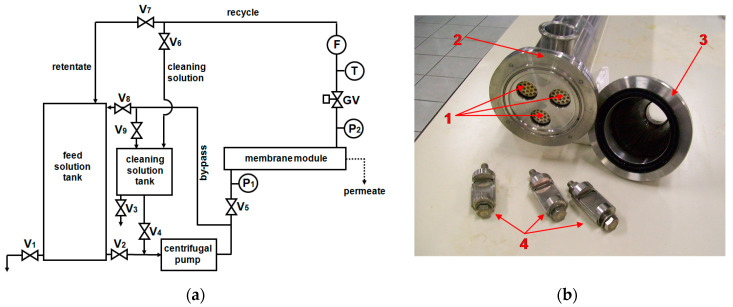
(**a**) Schematic of the plant used for MF tests: V1-9 ball valves; GV gate valve; P1,2 manometers, T thermometer; F flowmeter. (**b**) Membralox module: 1—ceramic multi-channel membranes, 2—stainless steel membrane housing, 3—module end-cup, 4—clamps.

Microfiltration of prefiltered OMW was performed in a batch operation mode with the plant schematically shown in Figure 1a, using three ceramic membranes (Membralox EP19-40, Pall Corp., Port Washington, NY, USA) arranged in parallel into a stainless steel housing (Figure 1b). The main properties of these membranes are shown in Table 2. 

The MF retentate was completely recycled to the feed tank while the clean permeate was continuously withdrawn to be used for RO test. As can be seen from Figure 1a, OMW is fed by the centrifugal pump to the membrane module with a velocity v = 3.9 m/s (calculated from the ratio between the feed flow rate measured by the flow meter, F, and the membrane channels cross-section) at an average pressure P = 2.3 bar, unless otherwise reported, measured by two manometers, P1 and P2, located before and after the membrane module, respectively. The permeate flow rate is simply evaluated by measuring with a graduated tank the time necessary to produce a given permeate volume. Permeate flux is then calculated from the ratio between the permeate flow rate and the overall filtration surface area. The temperature measured by the thermometer, T, is kept constant at 30 °C by a cooling device immersed into the feed tank. An electric immersion heater in the cleaning tank provides a rapid heating of the cleaning solutions (NaOH and/or NaOCl solutions) used to remove foulants from the membrane. 

The scheme of the RO plant is very similar to that of the MF plant shown in Figure 1a. The main differences are related to the feed pump (piston), the pressure control valve (globe valve), and the use of a variable frequency drive ‘inverter’ to control the feed flow rate, Q (speed pump). The experimental conditions adopted for RO experiments were: P = 30 bar (unless otherwise reported), Q = 1000 L/h, T = 25 °C. A small cell was used for preliminary tests with flat sheet membrane samples (surface 0.0066 m^2^) listed in Table 3. A cylindrical vessel was employed to house a spiral wound membrane module (SW30HR Dow-Filmtec, now DuPont, Wilmington, Deleware; 4” diameter, 40” length, membrane surface 7.9 m^2^) during the successive bench scale experiments. Preliminary tests with small flat membranes were carried out keeping the feed concentration constant, and continuously recycling both permeate and concentrate streams to the feed tank. Concentration tests with spiral wound module were performed in a batch operation mode, following the same procedure previously described for microfiltration of OMW. During both RO and MF experiments samples of different streams were collected for analysis. 

Electrical conductivity, pH and suspended solids content were measured according to Standard Methods [37]. COD was determined with the spectrophotometric method using Merck Spectroquant@ test kits (Merck KGaA, Darmstadt, Germany). The method is analogues to EPA 410.4, US Standard Methods 5220 D, and ISO 15705. Phenols were determined with Folin–Ciocalteu reagent [38].

## 3. Results

### 3.1. Feed Pretreatment and Microfiltration

**Figure 2:**
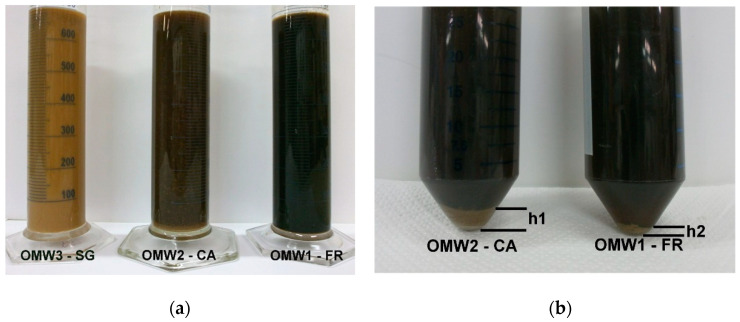
(**a**) Images of OMWs after settling and bag filtration. (**b**) Images of OMWs after centrifugation (h = TSS volume).

OMWs with quite different characteristics were received from three mills. In particular, the OMW3-SG was characterized by a very high load of suspended solids of small size with a negligible settling velocity and poorly retained by the filter bag as can be seen in Figure 2a, where the images of the three types of OMWs after settling and filtration treatment are reported for comparison. The darker color of samples OMW1-FR and OMW2-CA is connected to a high particle removal efficiency. However, even in these two cases (especially for OMW2-CA) the produced filtrates did not satisfy the requirements for the RO feed. This is apparent from the images of Figure 2b, where deposited solids after centrifugation (8000 rpm for 10 min) can be observed on the bottom of the centrifuge tube. Therefore, a post-filtration treatment with ceramic membrane with 0.2 µm pore size was employed for the removal of fine suspended solids and production of a suitable feed for RO [39]. The main physicochemical characteristics of the three types of OMW (after settling and bag filtration) fed to the microfiltration plant are reported in Table 4.

**Figure 3:**
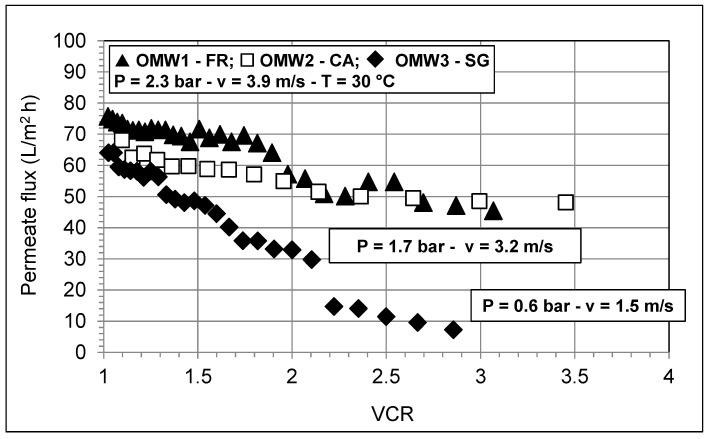
Permeate flux versus volume concentration ratio (VCR) during the MF tests with different OMWs.

Figure 3 refers to the microfiltration tests and shows the behavior of permeate flux as a function of volume concentration ratio, VCR (i.e., the ratio between the volume of the initial feed and the volume of the final concentrate) for the three different pretreated (settled and filtered) OMWs. Gentle heating at 30 °C makes the feed (especially the OMW3-SG) more fluid with consequent reduction of the friction loss along the plant and improved performance of the centrifugal pump. The permeate flux at the beginning of the MF tests appears very close for the three OMWs, while increasing the VCR, OMWs behave differently, especially OMW3-SG. As far as OMW1-FR and OMW2-CA are considered, the permeate flux first slightly decreases and then tends to level off. With OMW3-SG, which contains a relevant amount of suspended materials, a strong and almost proportional decline of permeate flux with increasing the VCR is observed. 

**Figure 4:**
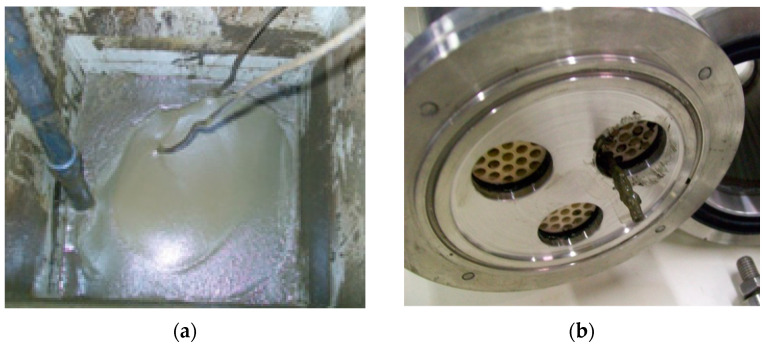
(**a**) OMW3-SG viscous concentrate after VCR = 2.1. (**b**) Removal of OMW3-SG muddy concentrate from the membrane channel with a metallic probe.

After a VCR = 2.1, the high viscosity of the concentrated OMW3-SG (Figure 4a) considerably reduces the performance (head and flow rate) of the centrifugal pump, thus the fluid velocity through the membrane channels is progressively lowered and membrane channels begin to plug. The permeate flux first falls and then continues the decrease slowly. Immediately after VCR = 2.85, a sudden increase of the pressure occurred and the pump reached its shut-off pressure. The test had to be stopped immediately and it was necessary to use a metal probe to unclog membrane channels (Figure 4b). 

Moreover, an intense membrane cleaning with NaOH solution (2% *w*/*w*) and NaOCl (500 ppm Cl) at 60 °C for at least 60 min was used to remove the deposit remaining on the membrane surface and into the membrane pores. By measuring pure water flux before (J_W,0_) and after (J_W,F_) OMW filtration, a flux recovery ratio FRR = (J_W,F_/J_W,0_)·100 very close to 100% was achieved, thus demonstrating the effectiveness of the cleaning procedure. The other two types of OMWs (1-FR and 2-CA) did not plug membrane channels but severely fouled the membrane. The pure water flux after MF was around 30% of the original membrane flux, but even in this case a FRR ≈ 100% was obtained after the cleaning with NaOH and NaOCl. 

The main physicochemical properties of feed (FD) and permeate (PR) samples collected at increasing VCR during the MF of the three types of OMW are listed in Table 5. Both pH and electrical conductivity of feed and permeate are substantially similar since dissolved ions pass through the pore of the membrane while a given retention is observed for COD due to the removal of suspended organic part, which contributes to this parameter. Phenol retentions seem to be high for the MF membrane, but according to previous literature findings [8], this fact can be ascribed to fouling, which may deeply alter the retention characteristics of membrane by itself. 

### 3.2. Nanofiltration and Reverse Osmosis

The results of NF/RO screening tests with small flat sheet membranes carried out by using the MF permeate of OMW2-CA as feed are reported in Table 6. Except for Desal DK, all the other membranes present very high solute retention. To obtain useful products from OMW such as purified water (permeate) and a polyphenols rich solution (concentrate), a membrane with the highest possible retention to salts, COD and phenols are required. Table 6 reveals that SW30HR membrane (DOW) completely meets these requirements. Therefore, this membrane in a spiral wound configuration was selected for successive bench-scale tests. 

**Figure 5:**
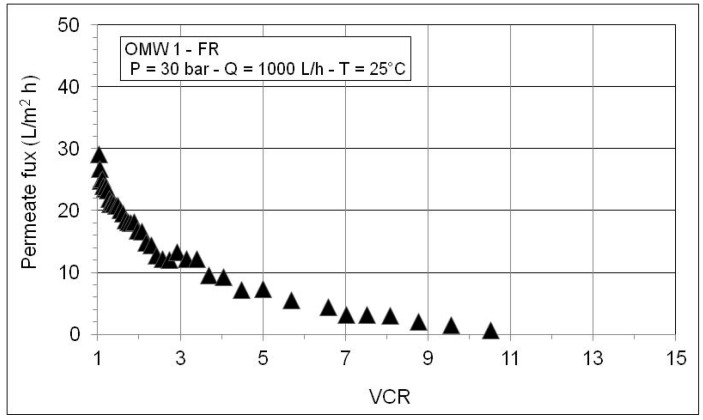
Permeate flux as a function of VCR during RO test with the MF permeate of OMW1-FR.

The results of the RO concentration test carried out with the MF permeate of OMW1-FR are shown in Figure 5. By increasing the VCR, the permeate flux decreases first rapidly and then slowly until reaching VCR = 10.5, a value (around 1 L/(m^2^∙h)) ca. 30 times lower than that of the initial flux (VCR = 1). The observed flux decline with increasing VCR can be ascribed to the increase of the osmotic pressure of the feed, as well as concentration polarization and fouling phenomena. 

**Figure 6:**
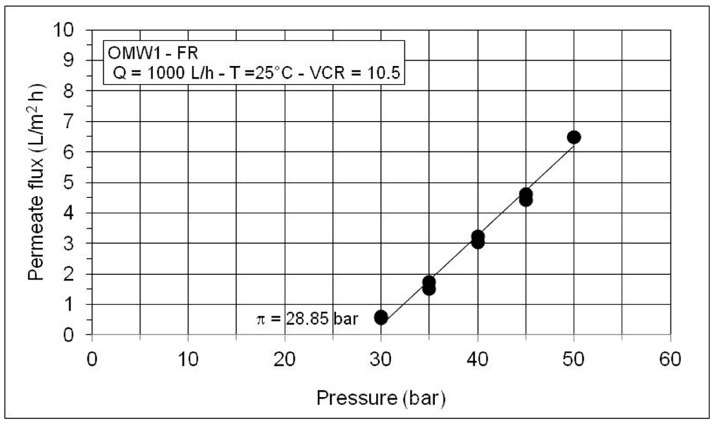
Permeate flux as a function of operating pressure at VCR = 10.5 (Feed: MF permeate of OMW1-FR).

**Figure 7:**
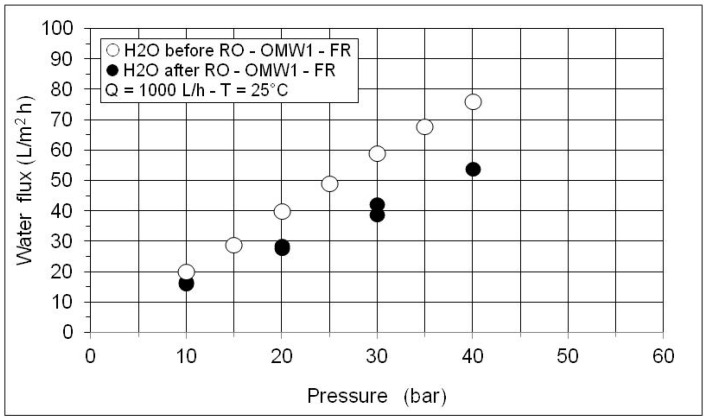
Pure water flux as a function of operating pressure before and after RO test with MF permeate of OMW1-FR.

The osmotic pressure of the concentrated solution (VCR = 10.5) can be estimated by measuring the permeate flux at increasing pressures and a constant feed concentration as shown in Figure 6. The differences in pure water flux (Figure 7) measured before and after OMW treatment are connected to the membrane fouling. Only a moderate cleaning with a NaOH solution (pH = 11) at 40°C was sufficient for eliminating fouling and achieving FRR around 100%. 

**Figure 8:**
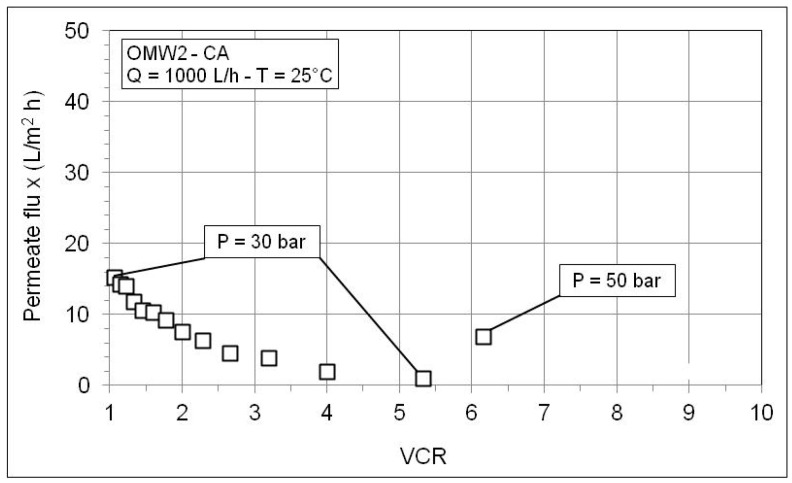
Permeate flux as a function of VCR during RO test with the MF permeate of OMW2-CA.

**Figure 9:**
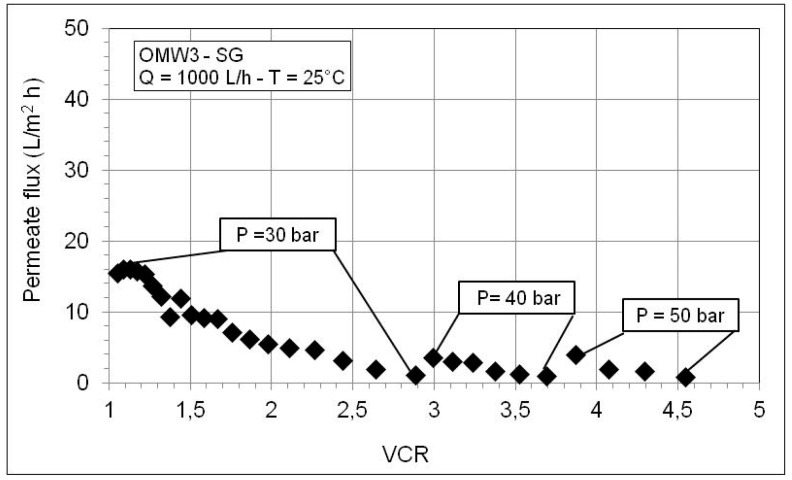
Permeate flux as a function of VCR during RO test with the MF permeate of OMW3-SG.

Figure 8 and Figure 9 show the behavior of the permeate flux as a function of VCR during the RO concentration of the permeates produced by microfiltration of OWM2-CA and OWM3-SG. The trends are similar to that shown in Figure 5. The permeate flux improves at higher pressure but continues to fall with the increase in the VCR. A worse membrane performance is observed according to the considerably higher solute content of these OMWs as shown in Table 7. Further inspection of Table 7 reveals high retention values for conductivity and COD and an excellent abatement of phytotoxic phenol fraction. As expected, the retention worsens with VCR and improves with the pressure (since water flux through the membrane increases with the pressure while the solute diffusion is independent of pressure).

**Figure 10:**
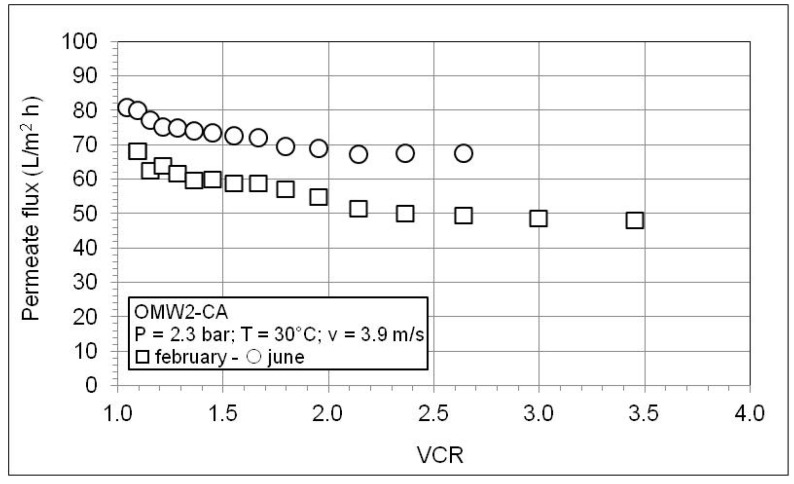
Effect of storage of OMW2-CA on the permeate flux as a function of VCR during MF test.

**Figure 11:**
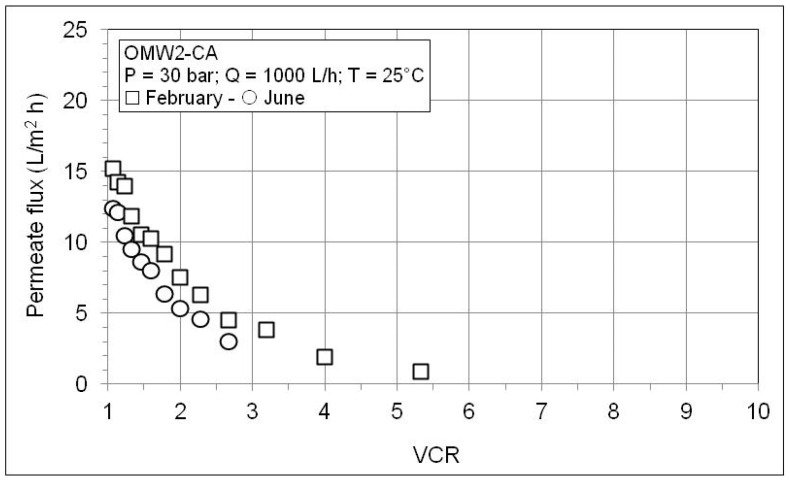
Effect of storage of OMW2-CA on the permeate flux as a function of VCR during RO test with the MF permeate of OMW2-CA.

The effect of OMW age on the performance of the membrane is shown in the following Figure 10 and Figure 11 and in Table 8. It is worth noticing that olive oil extraction is a seasonal operation whose duration is around 4–5 months during the winter. The amount of OMW is much higher than that of olive oil produced, and consequently very large plants are necessary for the treatment of all the wastewater generated daily, otherwise it must be stored. To obtain preliminary information on the influence of OMW storage/aging on the performance of the integrated membrane process, a given amount of OMW2-CA was allowed to rest for ca. 4 months. After this long settling period, the supernatant liquid was filtered through the usual filter bag and the resulting filtrate was sent to the MF plant. From the results reported in Figure 10, a given increase of the permeate flux of the stored OMW is observed. This increase is connected to a lower content of suspended material (TSS = 420 mg/L) due to the 4 months settling period. Conversely, only a moderate variation of the permeate flux during the RO experiments (Figure 11) occurs since the amount of dissolved solids does not practically change during the storage, as can be seen from physicochemical characterization results shown in Table 8. From the same table it is apparent that the storage period does not affect membrane retention.

## 4. Discussion

### 4.1. Pretreatment and Microfiltration

As reported in Table 1 and Table 3, OMW contains relevant concentrations of TSS. Therefore, any type of membrane process aimed at polyphenol recovery as well as water reuse needs a pretreatment to remove TSS. The removal of TSS is of crucial importance for the fouling control of the NF or RO process. 

Cassano et al. [12] pretreated the raw OMWs by using a commercial tubular MF membrane module (pore size 0.2 µm, polypropylene, 5.5 mm inner diameter). Then, UF polymeric membranes were used to produce a clear permeate to be fed to the nanofiltration unit. Nevertheless, for all the UF membranes, a flux decay was observed. Bazzarelli et al. [29] studied the change of pH to destabilize the solid suspension in OMW and they showed that a pretreatment based on MF or UF can be effective at removing the suspended solids. MF exhibits higher fluxes than UF and ceramic membranes showed the highest fluxes. In another interesting approach of MF by using polymeric hollow fiber membranes, a fouling control was attempted by the deposition on the membrane surface of a photoactive gel [40]. 

Garcia–Castello et al. [11] reported the performance of a 0.2 µm alumina membrane after several filtration runs. During each run, a consistent flux decay was observed and even after a cleaning procedure with 20 g/L NaOH at 40 °C the water flux of the virgin membrane was not fully recovered. 

In our work, we proposed the use of a ceramic MF membrane due to its high chemical and thermal stability during the cleaning procedures to restore its performance. We also observed an evident decay of the flux (Figure 3), but on the other hand, a chemical cleaning with alkaline agents combined with the use of a sufficiently high temperature (about 60 °C for at least 1 h) we restored the initial membrane performance. The effect of the temperature during the chemical cleaning was remarked in a recent work also by Fraga et al. [41], where the use of high permeability silicon carbide MF membranes was investigated. 

The MF ceramic membrane module tested with all the three OMWs was able to preserve the subsequent NF or RO spiral wound modules from plugging problems. Since MF can seriously suffer from plugging and fouling phenomena at high TSS, the use of chemically-resistant membranes seems to be essential, especially if the plant is designed to be used only seasonally. 

### 4.2. Nanofiltration and Reverse Osmosis

SW30HR membrane showed the best retention of COD and phenols among the tested NF and RO membranes. By increasing the VCR, the retention to phenols was always very high (>99.3%) and the retention to COD had generally been about 95%. The highest VCR obtained was limited by the increase of the osmotic pressure. As shown, at VCR = 10.5 the experimental osmotic pressure was approaching 29 bars. The electrical conductivity of pristine OMW1-FR was 5310 µS/cm, while for OMW2-CA and OMW3-SG, the electrical conductivities were very close, 13,940 µS/cm, 12,780 µS/cm, respectively (Table 4). Since the electrical conductivity is mainly related to the concentration of dissolved salts, with the OMW1-CA it was possible to achieve a higher VCR than for the other two OMWs. The different behavior between OMW2-CA and OMW3-SG during the RO concentration is therefore mainly related to the different level of organic compounds, considering that the ratio of COD between the OMW3-SG and OMW2-CA is about 2. A pressure increase seems to be beneficial to both the flux and the retention. 

### 4.3. Remarks

On the basis of our results and of the findings reported in the literature, integrated membrane processes are able to efficiently produce a polyphenols-rich concentrate. The recovery of polyphenols is very interesting, since they are valuable compounds that can be supplied to cosmetic and pharmaceutical industries. Nevertheless, the exploitation of polyphenols-rich streams is still facing some technological challenges related to the polyphenol fractionation. The main driver to develop processes for the treatment of OMW is the environmental pressure in order to limit the pollution related to their production and disposal. A clean water stream can be obtained when an RO process is considered as a final step. In the proposed integrated membrane process, the high retention of polyphenols can allow the separation of good quality water already after a first RO stage, which can be more easily accepted by a sewage depuration system since the residual COD is no longer related to the presence of polyphenols. The permeate water can be considered also for an internal reuse in the olive mill after and eventual refining treatment (a second RO stage or adsorption) as well as for irrigation purposes. The main process issues are related to controlling the fouling. Ceramic membranes have proved their suitability since they can withstand aggressive chemical cleaning procedures, and although their cost is still high compared to that of polymeric membranes, they can guarantee a longer lifetime. Since in many countries olive mills are still small enterprises, the investment costs for a membrane-based treatment plant can be more easily faced if there is the possibility of storing part of the OMWs generated during the milling season. In this work, we proved that the aging of the OMW does not critically affect the performance of the integrated membrane process. 

Pulido and Martinez-Ferez in their review [42] identified the control of fouling as one of the limits of membrane technologies applied to OMW. Another bottleneck for a wide field application of the integrated membrane process remains, related to the options available for either the disposal or chemical/energetic valorization of the concentrate stream. These options should be evaluated on the specific characteristics and constraints of the olive mill willing to apply membrane technology. 

## 5. Conclusions

MF/RO integrated membrane processes have been proposed for the treatment of OMW. The MF can be considered a suitable pretreatment for RO process since it provides a clean permeate, which does not cause plugging of the spiral wound element. RO separates dissolved substances from water, thus allowing the concentration of valuable products and produces water with a low salinity, COD, and phytotoxicity. Channel plugging and fouling of MF membrane represent a serious problem during the treatment of OWM, characterized by a high load of fine particles, which cannot be properly removed by simple settling or bag filtration. Therefore, ceramic membranes capable of withstanding hard cleaning agents are necessary. Membrane performance is not deeply affected by OWM aging and consequently, the wastewater may be treated gradually, without the need of large plants operating only a few months a year. This may involve important benefits connected to the reduction of investment costs and of bactericide solutions, which are necessary for a long-term storage of delicate RO membranes. The commercial RO membrane for seawater treatment, SW30 HR (Dow), showed a very high retention to polyphenols and dissolved species, which contribute to electrical conductivity. The increase of both osmotic pressure and organics concentration limited the maximum volume concentration ratio that could be achieved. 

## Figures and Tables

**Table 1:** Analytical characteristics of olive mill wastewater (OMWs) from the batch (BP), two-phase (2P) and three-phase (3P) processes and some integrated membrane processes proposed in literature.

Process	pH	EC	TSS	COD	Ph	Ch	Proposed Process	References
		(mS/cm)	(g/L)	(g/L)	(mg/L)	(g/L)		
BP	4.5–5		0.1–2.7	65.7–130	1.2–2.4%	2.2–4.5		[5]
	4.5	9.0	12%	−180.0	-	-		[21]
	4.5	-	8.0	47.8	3740	-	UF-NF	[22]
	4.7	-	7.8	59.1	4560	-		
2P	5.1	1.8	-	13.4	749	-	NF	[23]
	5.25	2.1	-	14.0	776	-		
	5.5	2.2	-	4.2	-	-	NF	[24]
	4.9	1.7	5.6	16.4	181	-	RO	[25]
	4.9	1.3	0.6%	7.8		-		[21]
3P	4.7–5.2	-	0.9–27.6	40–103.4	0.37–0.5%	1.5–4.7		[5]
	5.0	-	17.6		212	-	MF-NF-OD	[11]
	-	-	44	107.2	2640	12.3	UF-NF-RO	[26]
	5.4	7.9	6.6%	151.4	-	-		[21]
	5.13	5.08	11.7	16.5	850	13.1	UF-NF-RO	[27]

EC = Electrical conductivity; TSS = Total Suspended Solids; TOC = Total Organic Compounds; COD = Chemical Oxygen Demand; Ph = Polyphenols; Ch = Carbohydrates.

**Table 2:** Main properties of Pall–Membralox EP19-40 membrane used for MF tests.

Channel Diameter	4 mm
Number of Channels	19
Filtration Surface Area	0.24 m^2^
Length	1020 mm
Material	Ultrapure α-alumina (>99.7%)
Pore size of the inner layer	0.2 µm

**Table 3:** NF and RO membrane used during test cell experiments.

Membrane	Manufacturer	Minimum Rejection	Application
Desal AG	GE Power&Water	99.3% (NaCl)	Brackish water RO
Desal SC	GE Power&Water	98.5% (NaCl)	Brackish water RO
Desal DK	GE Power&Water,	98% (MgSO_4_)	NF
SW30 HR	DOW	99.6% (NaCl)	Seawater RO
SW30 ULE	DOW	99.55% (NaCl)	Seawater RO
BW30	DOW	99.0% (NaCl)	Brackish water RO
NF 90	DOW	97.0% (MgSO_4_), 85.0% (NaCl)	NF

**Table 4:** Physicochemical properties of OMWs fed to the microfiltration plant.

	pH (—)	TSS (mg/L)	COD (mg/L)	Conductivity (µS/cm)	Phenols (mg/L)
OMW1-FR	4.43	870	30,000	5310	2000
OMW2-CA	5.80	1700	66,500	13,940	1500
OMW3-SG	5.17	15,000	159,500	12,780	3300

**Table 5:** Main physicochemical properties of feed (FD) and permeate (PR) samples collected during the MF tests with three different types of OMW.

VCR	pH		Conductivity (µs/cm)			COD (mg/L)			Phenols (mg/L)		
	FD	PR	FD	PR	R (%)	FD	PR	R (%)	FD	PR	R (%)
OMW1-FR											
1.00	4.43	4.39	5310	5050	4.90	30,000	19,780	34.07	2000	1430	28.50
1.29	4.44	4.39	5450	5330	2.20						
1.82	4.44	4.4	5420	5290	2.40	33,425	20,450	38.82	3900	2820	27.69
3.07	4.5	4.41	5450	5260	3.49	38,700	23,100	40.31	4500	3200	28.89
OMW2-CA											
1.00	5.8	5.9	13,940	13,690	1.79	66,500	43,370	34.78	1500	996	33.60
1.22	5.79	5.88	13,600	13,350	1.84	70,889	40,990	42.18			
1.55	5.77	5.91	13,560	13,410	1.11	75,500	42,440	43.79	1590	1050	33.96
2.14	5.79	5.87	13,920	13,650	1.94	85,000	40,740	52.07			
3.45	5.81	5.9	13,990	13,500	3.50	95,000	42,850	54.89	1910	1200	37.17
OMW3-SG											
1.00	5.17	5.23	12,780	12,240	4.23	128,800	49,490	61.58	3300	1950	40.91
1.67	5.37	5.38	12,470	12,060	3.29	164,350	55,350	66.32			
2.86	5.37	5.39	12,470	12,050	3.37	209,000	64,590	69.10	4800	2820	41.25

**Table 6:** Results of the RO screening tests with different types of NF and RO membranes (P = 30 bar, T = 25 °C). Feed: MF permeate of OMW2-CA (Conductivity = 13,200 µS/cm; COD = 40,180 mg/L; Phenols = 1070 mg/L).

Membrane	Permeate Flux (L/m^2^h)	Conductivity		COD		Phenols	
		PR (µS/cm)	R (%)	PR (mg/L)	R (%)	PR (mg/L)	R (%)
Desal AG	7.00	138.6	98.95	2608	93.51	6.0	99.44
Desal SC	14.35	170.3	98.71	2326	94.21	9.1	99.15
Desal DK	60.61	1473	88.84	8249	79.47	112.8	89.46
SW30 HR	12.66	109.6	99.17	1736	95.68	2.9	99.73
SW30 ULE	12.04	151.8	98.85	2359	94.13	4.4	99.59
BW30	33.26	159.7	98.79	2387	94.06	9.0	99.16
NF 90	27.27	150.5	98.86	2668	93.36	7.5	99.30

**Table 7:** Main physicochemical properties of feed (FD) and permeate (PR) samples collected during RO test with the MF permeates of three different types of OMW.

VCR	P (bar)	pH		Conductivity (µS/cm)			COD (mg/L)			Phenols (mg/L)		
		FD	PR	FD	PR	R (%)	FD	PR	R (%)	FD	PR	R (%)
OMW1-FR												
1.00	30	4.48	3.79	5100	89.7	98.24	19,650	628	96.80	2900	6.0	99.79
1.96	30	4.58	3.61	8880	109.0	98.77	36,170	926	97.44			
6.56	30	4.68	3.83	20,900	253.0	98.79						
10.50	30			26,700	1382	94.82	140,120	2558	98.17	24300	28.0	99.88
OMW2-CA												
1.00	30	5.88	4.81	13,200	116.3	99.12	40,180	2010	95.00	1070	5.0	99.53
1.23	30	5.87	4.84	15,200	133.4	99.12	53,990	2231	95.87			
1.60	30	5.86	4.86	16,370	199.2	98.78	65,860	2495	96.21			
2.29	30	5.9	4.9	18,910	239.7	98.73	86,960	3840	95.58			
4.00	30	5.98	4.92	20,490	318.9	98.44	109,220	8855	91.89			
5.33	30	6.02	4.96	27,400	904.0	96.70	151,700	15,325	89.90	5600	57.3	98.98
6.15	50	6.12	4.89	30,200	702.0	97.68	191,350	4075	97.87	6420	24.2	99.62
OMW3-SG												
1.00	30	5.45	5.27	12,100	115.2	99.05	58,560	2933	94.99	2250	2.2	99.90
1.24	30			17,130	128.4	99.25	78,280	3189	95.93			
1.62	30			20,500	192.7	99.06	104,320	4543	95.65			
2.33	30	5.5	5.21	25,800	738.0	97.14	120,800	10,000	91.72			
2.74	30			29,000	1592	94.51	159,220	13,855	91.30	7500	54.0	99.28
3.62	40			33,800	1734	94.87	215,850	16,620	92.30			
4.57	50	5.59	5.16	36,100	3320	90.80	261,900	8720	96.67	9850	26.0	99.74

**Table 8:** Main physicochemical properties of feed (FD) and permeate (PR) samples collected during the RO test with “aged” OMW2-CA.

VCR	P (bar)	pH		Conductivity (µs/cm)			COD (mg/L)			Phenols (mg/L)		
		FD	PR	FD	PR	R (%)	FD	PR	R (%)	FD	PR	R (%)
1.00	30	5.91	4.82	13,500	117.4	99.13	40,200	2050	94.90	1025	5.2	99.49
1.14	30	5.87	4.84	14,200	135.2	99.05	44,500	2120	95.24			
1.33	30	5.93	4.85	15,300	198.7	98.70	55,450	2290	95.87			
1.60	30	5.94	4.87	16,450	240.1	98.54	67,800	2370	96.50			
2.00	30	5.89	4.83	17,930	303.8	98.31	84,300	3540	95.80	2320	11.0	99.53
2.67	30	5.93	4.90	19,800	318.4	98.39	98,650	5900	94.02			
